# Comparison of Effects of Chorionic Gonadotropin Alfa and Anastrozole on Sperm Retrieval Rate in Patients with Non-Mosaic Klinefelter Syndrome Following Microdissection Testicular Sperm Extraction

**DOI:** 10.3390/medicina61030467

**Published:** 2025-03-07

**Authors:** Eyyup Sabri Pelit, Yavuz Onur Danacıoğlu, Bülent Katı

**Affiliations:** 1 Department of Urology, Faculty of Medicine, Harran University, Sanliurfa 63300, Turkey; bulentkati@harran.edu.tr; 2 Clinic of Urology, Bakırköy Training and Research Hospital, University of Health Sciences, Istanbul 34668, Turkey; yavuz.danacioglu@saglik.gov.tr

**Keywords:** klinefelter syndrome, azoospermia, micro-tese

## Abstract

*Background and Objectives*: This study aimed to compare the effects of choriogonadotropin alfa and anastrozole treatments on the success of sperm retrieval in patients with Klinefelter syndrome (KS) undergoing micro-TESE at our clinic. *Materials and Methods*: We conducted a retrospective review of a cohort including patients with non-mosaic KS who underwent micro-TESE for fertility treatment at the Reproductive Medicine Center of our university hospital. This study included 43 patients who had not received exogenous testosterone therapy prior to or during the procedure. Before surgical sperm retrieval, all patients received either choriogonadotropin alfa or anastrozole treatment based on their preference. Micro-TESE was performed on all patients after three months of treatment. *Results:* The overall SRR in the cohort post-micro-TESE was found to be 32.6%. There was a significant increase in post-treatment testosterone levels compared to pre-treatment levels. Upon dividing patients into two groups based on whether sperm was successfully retrieved, we observed significant improvements in testosterone levels in both groups following treatment. In the group presenting with successful sperm retrieval, 28.6% of patients had received choriogonadotropin alfa, while 71.4% had received anastrozole. No statistically significant difference was found between treatment groups in terms of micro-TESE success. Both choriogonadotropin alfa and anastrozole treatments resulted in significant improvements in testosterone levels following treatment compared to pre-operative levels. Furthermore, in the choriogonadotropin alfa group, there were significant decreases in follicle-stimulating hormone and luteinizing hormone levels, as well as a significant increase in estradiol levels after treatment. Post-treatment E2 levels were significantly lower in the anastrozole group than in the choriogonadotropin alfa group (*p* = 0.032), while the mean testicular volume was statistically significantly lower in the choriogonadotropin alfa group. *Conclusions:* This study suggests that anastrozole treatment before micro-TESE in patients with KS yields more successful results in terms of the SRR compared to choriogonadotropin alfa treatment.

## 1. Introduction

Klinefelter syndrome (KS), also known as 47, XXY, is the most common chromosomal abnormality characterized by the presence of an extra X chromosome in the standard male karyotype [1]. It has a prevalence of 50 per 100,000 and is found in 14% of azoospermic patients [2]. Approximately 90% of patients with KS present with the 47, XXY karyotype, while 10% have mosaicism or, more rarely, X chromosome aneuploidy [3,4]. Due to its rarity, only 25% of adult males with KS are diagnosed, and diagnosis before puberty is uncommon [2]. Typical clinical manifestations of KS include bilateral symmetric, small, firm testes, gynecomastia, hypergonadotropic hypogonadism, and infertility. Genetic alterations in KS result in testicular hyalinization and widespread fibrosis of seminiferous tubules, impairing spermatogenic function and causing infertility [5,6].

Patients with KS are commonly reported to be infertile, and adolescents and young men with normal sperm counts in ejaculated semen are rare, with rates reported between 0% and 4% [7]. However, in patients with non-mosaic KS, focal spermatogenesis may be present. With advancements in microdissection testicular sperm extraction (micro-TESE), a technique used in assisted reproductive technologies, some patients with this condition can achieve biological fatherhood [6,8].

The chromosomal abnormalities in KS result in hypergonadotropic hypogonadism and androgen deficiency. Despite being highly stimulated by elevated circulating luteinizing hormone (LH) levels, Leydig cells in these patients are generally considered dysfunctional and are unable to produce adequate testosterone (T) [6,9]. While specific prognostic parameters have yet to be established, hormone levels and testicular volume have been proposed to have potential prognostic value for successful sperm retrieval in KS [10]. There is evidence suggesting that hormone replacement therapy can increase intratesticular testosterone levels, thereby improving sperm production and the sperm retrieval rate (SRR) in micro-TESE [11]. In clinical practice, human chorionic gonadotropin (hCG), anastrozole, and exogenous T treatments are commonly used to elevate intratesticular T levels [6,11,12].

The existing literature indicates that data on the contribution of choriogonadotropin alfa and anastrozole treatments to the SRR in patients with KS undergoing micro-TESE remain insufficient [6]. Therefore, this study aimed to compare the effects of these two treatment methods on SRR success in patients with KS undergoing micro-TESE at our clinic.

## 2. Materials and Methods

We conducted a retrospective analysis of a cohort including patients with non-mosaic KS who underwent micro-TESE for fertility treatment at the Reproductive Medicine Center of Harran University Medical Faculty Hospital. Informed consent was obtained from all participants. Chromosomal analyses of patients who received genetic counseling at our institution were confirmed using G-banding on cultured peripheral lymphocytes, following standard techniques. Each patient with non-mosaic KS was evaluated according to the World Health Organization guidelines, which included semen analysis of centrifuged samples performed at least twice on separate occasions [13]. Semen samples from all patients with KS were centrifuged at 3000× *g* for 30 min to confirm azoospermia.

The inclusion criteria for this study were as follows: (1) the absence of sperm in semen samples following two separate tests and (2) a confirmed 47, XXY karyotype. The exclusion criteria were (1) postnatal factors impairing testicular spermatogenic function, such as urinary infections, trauma, or orchitis; (2) mosaicism; or (3) previous or concurrent testosterone replacement therapy or other endocrine treatments, or a history of unsuccessful micro-TESE.

This study included 43 patients who had not received exogenous T therapy prior to or during micro-TESE between January 2017 and June 2024. This study was approved by the Ethics Committee of Harran University (approval number: HRÜ/24.08.02), and all patients provided written informed consent. Each patient with KS was given the option to receive either choriogonadotropin alfa (Ovitrelle 250 mcg) or anastrozole (Arimideks 1 mg) treatment prior to surgical sperm retrieval, without any recommendation regarding the potential benefits or drawbacks of either medication. The preoperative treatment choice was made individually by each patient. For 21 patients with KS, 250 IU of choriogonadotropin alfa was administered intramuscularly 2 or 3 times a week for three months without additional oral medication. The remaining 22 patients received daily oral administration of 1 mg anastrozole tablets for three months. Sex hormone levels, namely follicle-stimulating hormone (FSH), LH, total T, estradiol (E2), and prolactin (PRL), were measured every month. Semen analysis was reassessed before surgery. Testicular volume was measured via physical examination using an orchidometer, with the larger testis volume used for analysis.

After obtaining informed consent from all patients, the surgical procedure was performed under spinal anesthesia. Following surgical preparation and sterile draping, a 2–3 cm midline scrotal raphe incision was made. The testis was delivered through the incision after passing through the layers of dartos fascia and tunica vaginalis. A longitudinal incision was made in the avascular area of the tunica albuginea, and the testicular parenchyma was exposed by clamping the edges of the tunica albuginea. Dissection of the testicular parenchyma was performed under a microscope (Karl Zeiss, Oberkochen, Germany) at 18–22× magnification, selectively targeting enlarged and opaque tubules likely to contain sperm cells. If necessary, superficial and deep testicular areas were examined, and microdissection biopsies were performed by carefully excising the selected enlarged and opaque tubules using microsurgical forceps.

In the absence of visible enlarged tubules, two to three random microbiopsies were taken from the upper, middle, and lower regions of the testis. Testicular tissues obtained during surgery were minced and fragmented using a pair of sterile needles, and the presence of spermatozoa was examined under an inverted microscope at 250× magnification. The entire Petri dish was checked, and if no spermatozoa were observed, the entire tissue and buffered medium were left to settle in a conical tube. The supernatant was removed and centrifuged at 300 g for 5 min. The pellet was resuspended in 50 µL of buffered medium and re-examined for spermatozoa presence by the embryologist. The tunica albuginea was closed with a continuous, non-absorbable 5-0 nylon suture. After ensuring hemostasis, the layers were closed anatomically. Patients were discharged on postoperative day 1. In cases of complications, scrotal ultrasonography was performed. All micro-TESE procedures were conducted by the same surgeon (E.S.P.).

## 3. Statistical Analysis

Descriptive statistics for data included mean, standard deviation, median, minimum, maximum, frequency, and percentage values. The distribution of variables was assessed using Kolmogorov–Smirnov and Shapiro–Wilk tests. The independent-samples *t*-test was conducted to analyze normally distributed independent quantitative variables, while the Mann–Whitney U test was used for non-normally distributed independent quantitative variables. The paired-samples *t*-test and the Wilcoxon test were utilized for dependent quantitative variables. The chi-square test was applied to analyze independent qualitative variables, and Fisher’s exact test was used when chi-square test conditions were not met. All analyses were performed using SPSS version 27.0.

## 4. Results

Patients were divided into two groups based on the treatment they received before micro-TESE. There were 21 patients in the choriogonadotropin alfa group and 22 in the anastrozole group. The mean age of the 43 patients with KS in the entire cohort was 29.1 ± 4.9 years, and the mean testicular volume was 15.4 ± 3.4 cc. Table 1 shows pre-treatment levels of FSH, LH, T, E2, and PRL. In addition, as indicated in this table, the overall SRR for the cohort following micro-TESE was 32.6%.

After treatment, no significant changes were observed in FSH, LH, PRL, and E2 levels compared to pre-treatment levels (*p* > 0.05). However, there was a significant increase in T levels following treatment (*p* < 0.05) (Table 2).

Upon dividing the cohort into two groups based on whether sperm was retrieved, we observed significant improvements in post-treatment T levels compared to pre-treatment levels in both successful and unsuccessful sperm retrieval groups (*p* = 0.002 and *p* = 0.004, respectively). There were no differences between the two groups in terms of other sex hormone levels, patient age, duration of infertility, or testicular volume (*p* > 0.005). Among patients with successful sperm retrieval, 28.6% had received choriogonadotropin alfa treatment, while 71.4% had used anastrozole treatment. No statistically significant difference was found between the treatment choice and micro-TESE success (*p* = 0.065) (Table 3).

The evaluation of patients according to the treatment method used revealed significant improvements in post-treatment T levels compared to pre-treatment levels in both the choriogonadotropin alfa and anastrozole groups (*p* < 0.05 for both). In addition, the choriogonadotropin alfa group presented with significant decreases in FSH and LH levels and a significant increase in E2 levels following treatment (*p* = 0.007, *p* = 0.05, and *p* = 0.018, respectively). The post-treatment E2 levels of the anastrozole group were statistically significantly lower than those of the choriogonadotropin alfa group (*p* = 0.032), while the mean testicular volume was statistically significantly lower in the choriogonadotropin alfa group (*p* = 0.001). Sperm was successfully retrieved via micro-TESE in 19% of patients who had received choriogonadotropin alfa, compared to 45.5% of those in the anastrozole group (Table 4).

## 5. Discussion

Various studies in the literature have evaluated the effects of hormone replacement therapy on sperm retrieval in patients with KS. However, our study is the first to directly compare choriogonadotropin alfa and anastrozole treatments, which are commonly used in clinical practice. This comparison is crucial because hormone modulation has been proposed as a method to enhance the SRR in KS patients, yet there are limited data on the efficacy of different hormonal treatments. While previous studies have explored various hormonal protocols, there has been no direct comparison between these two widely used treatments in this specific patient population [6,11]. By evaluating the impact of choriogonadotropin alfa and anastrozole on SRRs, as well as their effects on T, FSH, and E2 levels, our study provides valuable insights into optimizing preoperative hormonal therapy for KS patients undergoing micro-TESE.

In patients with KS, one of the factors leading to azoospermia has been reported to be the early death of spermatocytes [10]. Spermatocytes enter meiosis to develop into sperm; however, this process is disrupted in patients with KS, causing spermatocyte degeneration during the embryonic stage. The reduced number of spermatocytes results in the abnormal differentiation of Leydig cells, leading to T deficiency [6,7,14].

Preoperative hormonal treatments are used to increase endogenous T levels and have been associated with higher SRRs following micro-TESE in patients with KS [15]. Choriogonadotropin alfa, which is an analog of the gonadotropin LH, is one of the primary treatments used for this purpose, as it stimulates testosterone production by Leydig cells. Other medications, such as anastrozole and testolactone, aim to increase effective concentrations of endogenous testosterone by inhibiting its conversion to E2 via aromatase [11]. hCG acts as a substitute for LH, stimulating Leydig cells in the testes to produce testosterone. Medications like anastrozole and testolactone enhance the concentration of endogenous testosterone by inhibiting its conversion to estradiol through the action of aromatase. These hormonal treatments are commonly used to boost the body’s natural testosterone production, which has been linked to improved sperm retrieval outcomes for men with Klinefelter syndrome (KS) undergoing micro-TESE procedures. Several factors have been explored to predict the success of sperm retrieval with TESE, including age, testicular volume, sexual hormone levels (serum testosterone, FSH, and LH), and even the origin of the additional X-chromosome [16,17,18]. However, no definitive parameter has yet been identified to predict successful surgical sperm retrieval in patients with non-mosaic KS before surgery [6].

Both LH and choriogonadotropin alfa share the same LH/choriogonadotropin receptor and can stimulate testicular steroidogenesis [19]. In vitro, choriogonadotropin alfa has higher activity than LH in terms of cyclic adenosine monophosphate production. The T response can indicate the status of testicular function; therefore, measuring plasma T after choriogonadotropin alfa stimulation could be useful in predicting whether patients with KS have a fully functional spermatogenic system [6,7,8,9,10,11,12,13,14,15,16,17,18,19,20]. Guo et al. demonstrated that patients with KS with larger testicular volumes and higher pre-treatment T levels responded better to choriogonadotropin alfa and were more likely to achieve sperm retrieval via micro-TESE [6]. In our study, the SRR was lower in the choriogonadotropin alfa group than in the anastrozole group. However, the choriogonadotropin alfa group presented with a statistically lower mean testicular volume and lower pre-treatment T levels, although not statistically significant. This could explain why, despite significant increases in T and decreases in FSH, the choriogonadotropin alfa group had a lower SRR than the anastrozole group. Furthermore, there is evidence in the literature suggesting that aromatase inhibitors may be more effective in improving sperm retrieval success [8]. While comparing different hormone replacement therapies, it has been observed that aromatase inhibitors significantly reduce E2 levels despite higher FSH, LH, and T/E2 values [8]. In our study, although the anastrozole group did not achieve a significant reduction in E2, the post-treatment E2 levels of this group were lower compared to the choriogonadotropin alfa group.

Ramasamy et al. examined different hormone therapy options, including hCG, before micro-TESE in patients with KS and found that these therapies did not result in significant differences in the SRR; however, the authors reported an SRR of 86% in those with normal baseline T levels [21]. Regarding micro-TESE outcomes, the SRR ranges from approximately 27 to 63% in patients with non-obstructive azoospermia without genetic abnormalities, while this rate is reported to be around 30–57% in those with KS [7]. Guo et al., who conducted one of the largest series studies in the literature, detected an SRR of 43.4% [6]. In our study, the SRR was 19% in patients treated with choriogonadotropin alfa, 45.5% in those treated with anastrozole, and 32.6% for the entire cohort, consistent with the literature.

The overall SRR of 32.6% in our study is consistent with previously reported SRRs for micro-TESE in KS patients, which typically range from 30% to 57% [6,8]. Our comparison between choriogonadotropin alfa and anastrozole aligns with prior studies indicating that hormonal pre-treatment may enhance SRR [11,15]. Notably, the higher SRR observed in the anastrozole group (45.5%) compared to the choriogonadotropin alfa group (19%) suggests that aromatase inhibition may offer a more favorable approach in KS patients. This finding is consistent with reports emphasizing the potential benefits of anastrozole in increasing intratesticular testosterone while lowering estradiol levels, thereby improving spermatogenic outcomes [9].

This study was the first in the literature to compare the success of choriogonadotropin alfa and anastrozole treatments before micro-TESE in patients with KS. However, it had certain limitations, with the primary limitations being the retrospective design and non-randomized selection of treatment options. In addition, the choice of treatment regimen was left to the patient’s decision, which may have introduced bias. Lastly, the absence of a control group prevented the evaluation of the SRR in patients who did not receive preoperative hormone therapy.

## 6. Conclusions

Anastrozole treatment before micro-TESE was found to be more successful than choriogonadotropin alfa in terms of the SRR in patients with KS. We consider that higher post-treatment T levels and lower E2 levels contribute to more successful sperm retrieval. Despite conflicting results in the literature concerning the prediction of sperm retrieval success, it is evident that increasing T levels before the procedure improves the SRR in patients with KS. Our study may guide physicians in choosing treatment regimens to increase T levels in patients with KS who plan to undergo micro-TESE. However, more extensive randomized controlled trials are needed to support our findings.

## Figures and Tables

**Table 1:** Baseline and post-treatment characteristics of patients.

		Min–Max	Median	Mean ± SD/n-%
Age		21.0–43.0	29.0	29.1 ± 4.9
Infertility duration		1.0–17.0	5.0	5.8 ± 3.3
**FSH**				
Pre-treatment		1.1–90.6	43.3	45.8 ± 17.7
Post-treatment		2.7–104.6	37.0	39.4 ± 21.3
**LH**				
Pre-treatment		0.6–39.0	23.6	23.5 ± 8.4
Post-treatment		1.0–43.7	20.0	20.8 ± 10.2
**Testosterone**				
Pre-treatment		38.4–523.9	152.0	189.6 ± 103.1
Post-treatment		20.0–710.2	260.9	279.9 ± 132.6
**Prolactin**				
Pre-treatment		4.7–19.8	9.4	10.0 ± 3.2
Post-treatment		4.2–32.6	9.8	10.3 ± 4.8
**Estradiol**				
Pre-treatment		7.5–58.6	31.3	30.3 ± 11.9
Post-treatment		11.8–94.4	30.6	35.4 ± 17.5
Testicular volume (cc)		10.0–23.0	16.0	15.4 ± 3.4
Treatment	Choriogonadotropin			21 48.8%
	Anastrazole			22 51.2%
TESE	(−)			29 67.4%
	(+)			14 32.6%

SD: standard deviation, FSH: follicle-stimulating hormone, LH: luteinizing hormone, TESE: testicular sperm extraction.

**Table 2:** Comparison of pre- and post-treatment hormone levels.

	Pre-Treatment		Post-Treatment		*p*
	Mean ± SD	Median	Mean ± SD	Median	
FSH	45.8 ± 17.7	43.3	39.4 ± 21.3	37.0	0.080 ^W^
LH	23.5 ± 8.4	23.6	20.8 ± 10.2	20.0	0.051 ^P^
Testosterone	189.6 ± 103.1	152.0	279.9 ± 132.6	260.9	**0.000** ^W^
Prolactin	10.0 ± 3.2	9.4	10.3 ± 4.8	9.8	0.822 ^W^
Estradiol	30.3 ± 11.9	31.3	35.4 ± 17.5	30.6	0.231 ^P^

^P^: Paired-samples *t*-test, ^W^: Wilcoxon test. SD: standard deviation, FSH: follicle-stimulating hormone, LH: luteinizing hormone.

**Table 3:** Comparison of pre- and post-treatment parameters according to the success of TESE.

		TESE (−)		TESE (+)		*p*
		Mean ± SD/n-%	Median	Mean ± SD/n-%	Median	
Age		29.4 ± 5.3	29.0	28.6 ± 4.0	27.5	0.601 ^t^
Infertility duration		5.8 ± 3.7	5.0	5.7 ± 2.3	5.5	0.824 ^m^
**FSH**						
Pre-treatment		47.1 ± 19.3	43.7	43.0 ± 14.2	39.9	0.437 ^m^
Post-treatment		40.3 ± 21.7	37.0	37.6 ± 21.2	36.4	0.766 ^m^
Intra-group change *p*		0.082 ^W^		0.593 ^W^		
**LH**						
Pre-treatment		24.0 ± 9.0	23.4	22.5 ± 7.2	24.1	0.590 ^t^
Post-treatment		21.1 ± 10.4	20.1	20.2 ± 10.2	19.8	0.787 ^t^
Intra-group change *p*		0.088 ^P^		0.361 ^P^		
**Testosterone**						
Pre-treatment		196.4 ± 109.3	170.1	175.4 ± 91.2	141.3	0.452 ^m^
Post-treatment		278.0 ± 143.6	255.0	283.8 ± 111.1	275.4	0.437 ^m^
Intra-group change *p*		**0.002** ^W^		**0.004** ^W^		
**Prolactin**						
Pre-treatment		10.2 ± 3.5	9.8	9.4 ± 2.7	9.0	0.659 ^m^
Post-treatment		10.2 ± 5.0	9.8	10.5 ± 4.6	9.0	0.948 ^m^
Intra-group change *p*		0.417 ^W^		0.279 ^W^		
**Estradiol**						
Pre-treatment		30.5 ± 12.3	31.3	29.9 ± 11.7	31.5	0.959 ^t^
Post-treatment		35.3 ± 19.4	30.0	35.8 ± 13.5	36.3	0.613 ^t^
Intra-group change *p*		0.339 ^W^		0.552 ^W^		
Testicular volume (cc)		15.2 ± 3.5	15.0	15.9 ± 3.3	16.0	0.539 ^m^
Treatment	Choriogonadotropin	17 58.6%		4 28.6%		0.065
	Anastrozole	12 41.4%		10 71.4%		

^t^ Independent-samples *t*-test, ^m^ Mann–Whitney U test, ^P^ paired-samples *t*-test, ^W^ Wilcoxon test. SD: standard deviation, FSH: follicle-stimulating hormone, LH: luteinizing hormone, TESE: testicular sperm extraction.

**Table 4:** Comparison of pre- and post-treatment parameters according to hormone replacement therapy.

		Choriogonadotropin Alfa (n = 21)		Anastrozole (n = 22)		*p*
		Mean ± SD/n-%	Median	Mean ± SD/n-%	Median	
Age		29.6 ± 5.7	29.0	28.7 ± 4.0	28.5	0.535 ^t^
Infertility duration		6.3 ± 3.6	5.0	5.3 ± 2.9	5.0	0.391 ^m^
**FSH**						
Pre-treatment		45.4 ± 19.5	43.7	46.1 ± 16.4	40.7	0.846 ^m^
Post-treatment		33.7 ± 21.7	30.8	44.9 ± 19.9	41.0	0.068 ^m^
Intra-group change *p*		**0.007** ^W^		0.385 ^W^		
**LH**						
Pre-treatment		24.2 ± 9.1	24.5	22.8 ± 7.7	22.5	0.584 ^t^
Post-treatment		19.6 ± 12.4	18.9	22.0 ± 7.7	21.0	0.440 ^t^
Intra-group change *p*		**0.050** ^P^		0.591 ^P^		
**Testosterone**						
Pre-treatment		158.8 ± 83.1	143.1	218.9 ± 113.4	183.5	0.068 ^m^
Post-treatment		260.6 ± 142.3	243.7	298.3 ± 123.1	278.5	0.120 ^m^
Intra-group change *p*		**0.003** ^W^		**0.001** ^W^		
**Prolactin**						
Pre-treatment		8.9 ± 2.4	9.3	11.0 ± 3.6	11.0	0.063 ^m^
Post-treatment		10.1 ± 5.7	9.2	10.5 ± 4.0	10.0	0.552 ^m^
Intra-group change *p*		0.664 ^W^		0.532 ^W^		
**Estradiol**						
Pre-treatment		29.6 ± 10.7	31.5	31.0 ± 13.2	31.1	0.717 ^t^
Post-treatment		41.2 ± 19.4	40.9	29.9 ± 13.7	27.7	**0.032** ^t^
Intra-group change *p*		**0.018** ^P^		0.602 ^P^		
Testicular volume (cc)		13.8 ± 3.2	14.0	16.9 ± 2.8	18.0	**0.001** ^m^
TESE	(−)	17 81.0%		12 54.5%		0.065
	(+)	4 19.0%		10 45.5%		

^t^ Independent-samples *t*-test, ^m^ Mann–Whitney U test, ^P^ paired-samples *t*-test, ^W^ Wilcoxon test. SD: standard deviation, FSH: follicle-stimulating hormone, LH: luteinizing hormone, TESE: testicular sperm extraction.

## Data Availability

Our study data is unavailable due to privacy and ethical restrictions.
